# Phytotoxic effects of methylene blue dye on mustard (*Brassica juncea* L.): insights from germination bioassays and multivariate analysis

**DOI:** 10.1039/d6ra02447e

**Published:** 2026-05-21

**Authors:** Lopamudra Subudhi, Alok Kumar Panda, Shibani Mohapatra

**Affiliations:** a Centre for Biotechnology (CBT), Siksha ‘O’ Anusandhan (Deemed to be University) Bhubaneswar-751003 India shibanimohapatra@soa.ac.in; b Environmental Science Laboratory, School of Applied Sciences, Kalinga Institute of Industrial Technology (Deemed to be University) Bhubaneswar-751024 India; c Centre for Water Research and Climate Change, Kalinga Institute of Industrial Technology (Deemed to be University) Bhubaneswar 751024 Odisha India

## Abstract

Methylene blue (MB), a commonly employed cationic dye, contaminates aquatic and terrestrial ecosystems. Although its presence in polluted environments has been documented, little is known about its phytotoxicity to terrestrial agricultural crops. This study was conducted to assess the effect of MB on the germination and seedling development of a major oil crop, mustard (*Brassica juncea* L.), in a controlled hydroponic system. MB was found to significantly affect germination and seedling development. This effect was concentration-dependent. Germination was reduced by 50% compared to the control at higher concentrations. Similarly, root growth, shoot growth, seedling vigour index, and biomass were significantly reduced. On the other hand, relative toxicity and phytotoxicity increased substantially. To move beyond descriptive phenotyping, a predictive multivariate statistical analysis was employed to assess correlations between variables and establish physiological tipping points. Principal component analysis, hierarchical cluster analysis, and heatmap correlation analysis revealed a strong negative correlation between MB concentration and growth. Toxicity increased significantly with increasing MB concentration. This study revealed that MB significantly affects germination and seedling development and poses a potential risk to agricultural ecosystems, particularly during the early stages of crop establishment. Furthermore, by statistically identifying radicle elongation and seedling vigour as the most sensitive parameters, this study establishes them as reliable, early-warning biomarkers for agricultural risk assessment. Further field studies are needed to better understand its long-term impact on plant growth and productivity.

## Abstract 2

Methylene blue induced concentration-dependent phytotoxicity in mustard, with higher doses severely reducing germination, seedling vigor, root–shoot growth and biomass; PCA, clustering and heatmap validated distinct toxicity patterns.

## Graphical abstract

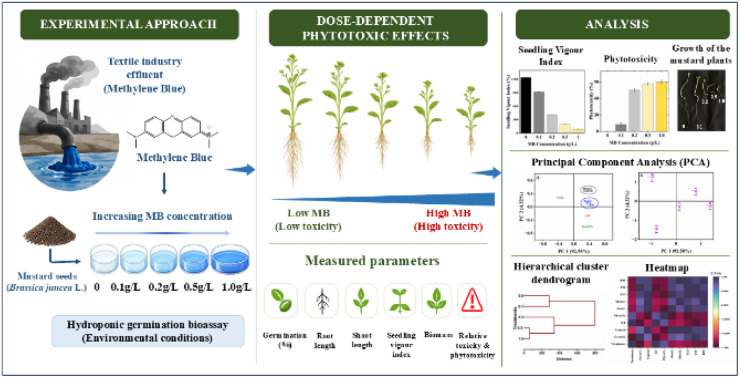

## Introduction

Synthetic dyes are widely used across industries such as textiles, clothing, plastics, rubber, leather, cosmetics, and pulp and paper. They are known as persistent pollutants in the environment and are classified as toxic, carcinogenic, and teratogenic agents that pollute aquatic environments.^1^ Among various industrial sectors, the textile industry is one of the major dye polluters, accounting for nearly 54% of total dye effluent discharge.^2^ At present, over 10 000 types of dyes are produced each year, which can be classified into three main categories: non-ionic dyes, cationic dyes, and anionic dyes, with a total production of over 1 × 10^6^ tons.^3^ It is estimated that about 10–15% of the dyes are lost during the manufacturing process and end up in water bodies. The wastewater generated from textile production is typically highly colored, hot, with a variable pH, high chemical oxygen demand, and high salinity.^4^ Synthetic dyes are found throughout the textile manufacturing process (*e.g.*, aniline blue, alcian blue, basic fuchsin, MB, crystal violet, toluidine blue, and Congo red).^5^ These compounds can persist in aquatic environments, reduce light penetration, impair photosynthesis, and disrupt aquatic food chains. Though many research works have been conducted on the pollution of water systems with dyes, little emphasis is given to the phytotoxic effects of synthetic dyes on agricultural crops, especially at the early stages of their development.^6^

Methylene Blue (MB) is one of the most used cationic dyes in the textile industry. MB has the chemical name of methylthionine chloride. It is a heterocyclic aromatic thiazine dye. It has applications in textile processing, biological staining, and medicine.^7,8^ MB is a heterocyclic aromatic colorant used in both the chemical and biological industries, as well as in other textile applications and as a medicine. Even at low concentrations, MB can cause prolonged toxicity in aquatic systems, primarily due to its residual properties. Therefore, wastewater from the textile industry may contain excessive amounts of colored waste from improperly treated operations prior to entering an aquatic environment, leading to elevated chemical oxygen demand (COD) within the aquatic system.^3^ Exposure to MB has been reported to lead to severe side effects such as headaches, nausea, and increased blood pressure.^9^ Though the toxicity of MB has been extensively studied in aquatic organisms and microorganisms, its effects on seed germination and seedling development have been less well explored, especially in crops.^5,7^

Controlled hydroponic bioassays have been widely used in recent literature to assess the phytotoxicity of various environmental contaminants. For example, hydroponic experiments showed that methylene blue is highly toxic to rice seedlings, resulting in significant reductions in relative growth rate and water-use efficiency.^5^ Similar concentration-dependent inhibitory effects on seed germination, root and shoot development, and seedling vigour have been observed in crops like red amaranth exposed to various loom synthetic dye effluents.^10^ Hydroponic systems have been widely used to evaluate the phytotoxicity of other industrial and agricultural pollutants, in addition to textile dyes. High levels of phenolic compounds and organic contaminants from wastewater have been shown to completely inhibit the germination of maize and tomato seeds^11^ and significantly inhibit the root elongation of crops such as lettuce^12^ and ryegrass.^13^ Similarly, such hydroponic systems have been successfully used to quantify the adverse effects of heavy metals, such as copper, lead, and zinc, and of agricultural pesticides on plant physiology. These studies reported severe physiological disruptions, including reduced biomass, inhibited nutrient uptake, impaired stem elongation, and decreased chlorophyll and protein content in various species, including wheat,^14^ emergent wetland vegetation,^15^ and Indian mustard.^16^ Seed germination and seedling emergence are highly sensitive bioindicators of environmental stress and have been widely used as rapid bioassays for phytotoxicity analysis.^17^ Mustard (*Brassica juncea* L.), an important oilseed crop in India, was chosen for the current study owing to its agricultural importance and known sensitivity to environmental pollutants.^18^ Physiological differences have been known to directly affect mustard yield and quality, thereby making the early growth stages highly susceptible to toxic stress. Mustard is also used as a model organism in phytotoxicity tests as well as environmental stress biology because of its fast germination, sensitivity to pollutants, and well-understood physiological responses.^19^ Using these well-established and sensitive methods, the present study employs a hydroponic system under controlled conditions to broadly evaluate the phytotoxic mechanisms of MB.

As discussed earlier, methylene blue (MB) is known for its environmental persistence and aquatic toxicity; however, little attention has been paid to its phytotoxicity on terrestrial agricultural crops during the early stages of development. There is a critical knowledge gap about the specific vulnerabilities of dicotyledonous oilseed crops to dye-contaminated irrigation water. The present work is novel because it goes beyond the classical single-parameter descriptive assays and uses a rigorous and predictive multivariate statistical framework (PCA, HCA, and heatmap analysis) in combination with non-linear dose–response modeling (EC_50_, IC_50_, and LD_50_). This comprehensive approach not only estimates the toxicity thresholds of MB on *Brassica juncea* L. but also identifies the specific interlinked physiological biomarkers, including radicle elongation and the seedling vigour index, that are most susceptible to dye-induced stress, thus providing a powerful diagnostic tool for agricultural risk assessment.

## Materials and methods

### Chemical reagents

**Fig. 1:**
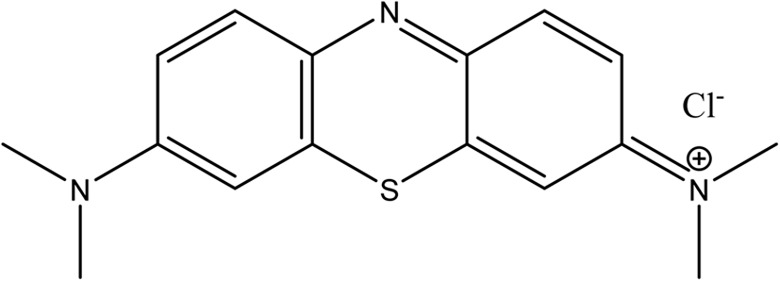
Molecular structure of MB dye. The image displays the two-dimensional chemical structure of methylene blue (C_16_H_18_ClN_3_S). It illustrates the central planar thiazine ring system, flanked by two dimethylamine groups, with a localized positive charge on the nitrogen–sulfur heterocyclic ring balanced by a chloride counterion.

MB is a synthetic cationic thiazine dye that was purchased from Sisco Research Laboratories Pvt. Ltd, Maharashtra, India. The molecular formula of MB is: C_16_H_18_ClN_3_S·3H_2_O, which has a molecular weight of 373.90 g mol^−1^ (Fig. 1). Stock solutions were prepared in distilled water and diluted to the required experimental concentration using appropriate dilution techniques.

### Collection of seed material

Mustard seeds were collected from a reputable local seed supplier and used as the test plant material for germination and root elongation. Before the experiment, the seeds were thoroughly screened to eliminate any that were damaged or malformed. Only healthy seeds with similar characteristics were selected for the experiment.

### Phytotoxicity assay

The mustard seeds were soaked in 1% sodium hypochlorite (NaClO) for 10 minutes to eliminate the chance of biological contamination. After the sodium hypochlorite was removed by washing the seeds three times with distilled water, the seeds were soaked in distilled water for 1 hour. Each germination test consisted of 3 repetitions. In all the sterile Petri plates had only one layer of tissue paper covering the bottom, 6 mL of each respective MB treatment solution was added onto the tissue paper, then 20 seeds per Petri plate with an approximate distance of 1 cm between seeds so that they would not overlap and each would have an equal opportunity to grow. Petri dishes were sealed and transferred to a dark growth chamber set at room temperature for 48 hours of incubation, then placed in an environmental condition and maintained a 12 hours light and dark cycle followed by a five-day period. During the incubation period, all three test runs were kept moist with the appropriate amount of moisture from the treatment solutions on the tissue paper. After the incubation period, the seedling growth parameters were measured.

### Parameters studied

The seedling growth parameters, such as germination percentage, germination speed, germination index, seedling vigour index, and relative toxicity, were measured. Morphological responses like length of shoots/roots, fresh and dry weight of mustard at various concentrations of MB in order to determine its phytotoxicity.^10^

#### Germination percentage (GER)

To determine the percentage of seedlings according to the following formula: (eqn (1))^20^1
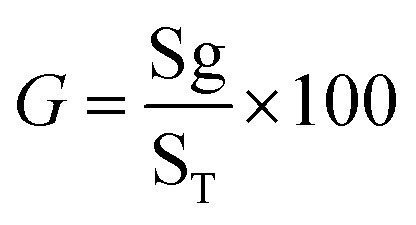
here, *G* = % germination, Sg = number of seeds germinated, and S_T_ = total number of seeds plated for the test.

#### Germination energy

The total germination percentage after three days.^21^

#### Germination capacity

The total germination percentage after seven days.^21^

#### Germination speed

Germination speed was measured by using the formula: (eqn (2))^22^2
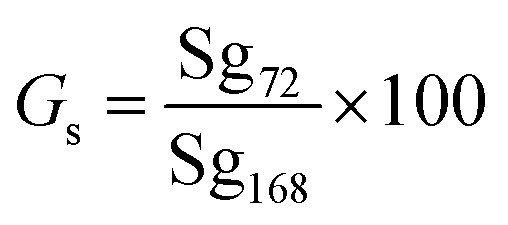
*G*_s_ = germination speed (%), Sg_72_ = number of seeds germinated after 72 hours, Sg_168_ = total number of seeds germinated after 168 hours, 100 = conversion factor for percentage. Germination speed was calculated as the percentage of seeds germinated at 72 h relative to the final germination count at 168 h.

#### Germination index (GI)

The germination index (GI) was calculated using the following formula: (eqn (3)).^23^3
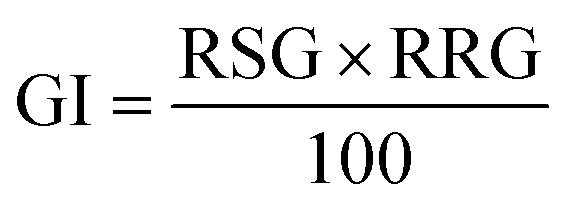
here, RSG is the relative seed germination, and RRG represents the relative root growth. RSG and RRG are calculated as follows: (eqn (4))4
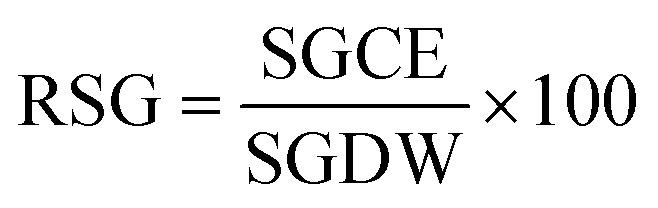
here, SGCE is the number of seeds germinated in the test sample or contaminated sample, and SGDW is the number of seeds germinated in the control or distilled water: (eqn (5))5
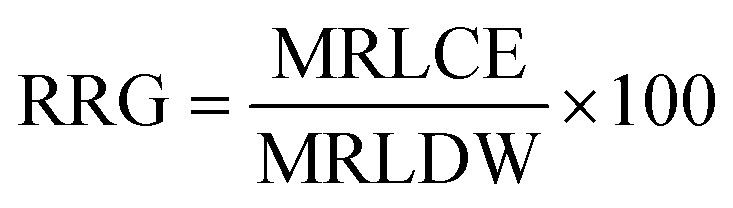
here, MRLCE is the mean root length in the test sample, and MRLDW is the mean root length in the control.

#### Relative toxicity (RT)

The relative toxicity of MB on the germination of mustard seeds and the growth of the seedling was calculated using the following formula: (eqn (6)).^24^6
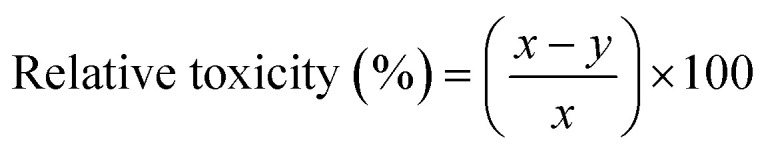
here, *x* = germination percentage or seedling length in the control at a specific hour of incubation, and *y* = germination percentage or seedling length in the presence of dye at the same hour of incubation.

#### Phytotoxicity (%)

The phytotoxicity was calculated using the formula of eqn (7).^25^7
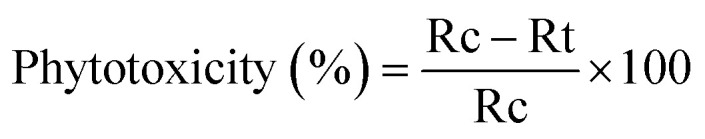
here, Rc = radicle length of control and Rt = radicle length of test.

#### Seedling vigour index

Vigour index of the seedlings was calculated using the formula proposed by eqn (8).^26^

8 Vigour index = *G* × *L*

here, *G*= germination (%) and *L* = seedling length.

#### Radicle and plumule length

The length of the radicle and the plumule of the seedlings was measured individually using the standard centimeter scale and expressed in centimeters (cm).

### Determination of fresh and dry weight

After harvesting, the length of the seed root buds was recorded using a centimeter scale. The root buds were cleaned thoroughly using distilled water and dried to remove moisture from their surfaces. The fresh weight (FW) of the seeds was recorded using an electronic balance. After recording the fresh weight, the seeds were subjected to heat treatment using an oven at 60 °C for 10 hours. The drying process continued until a constant weight was attained. The dry weight (DW) of the seeds was recorded after cooling the seeds to room temperature using a desiccator.

### Statistical analysis

All the parameters investigated, including germination percentage, germination speed, germination index, seedling vigour index, relative toxicity, phytotoxicity, root length, shoot length, dry weight, fresh weight, and the mean values of the seedling traits, were performed in triplicate, and the results were expressed as mean values ± standard deviation. The analysis was done using OriginPro 2024 software, USA. Multivariate analysis, including principal component analysis, hierarchical clustering, and a heatmap of Pearson's correlation, was performed to understand the relationships among the variables and the effects of treatment. Principal component analysis was done using standardized values, and cluster analysis was done using Euclidean distance. Nonlinear regression analysis was performed to determine the values of EC_50_, IC_50_, and LD_50_.^27^

### Dose–response analysis

The dose–response relationships were analyzed using the nonlinear regression function of the OriginPro program (OriginLab Corporation, USA). Separate fitting curves were obtained for the concentration-dependent responses of germination percentage, germination speed, seedling vigour index (SVI), root length, shoot length, and phytotoxicity. EC_50_ values were obtained for the parameters related to germination performance, IC_50_ for growth inhibition-related parameters, and LD_50_ for the phytotoxicity-related responses. The nonlinear regression model was used to estimate the concentration required for a 50% effect from the sigmoidal curve's midpoint. Model adequacy was assessed from the regression output generated by the nonlinear regression function in OriginPro. This includes the *R*^2^ value, which measures the model's goodness-of-fit. In addition, the standard fitting error should be reported if available. Furthermore, the 95% confidence intervals of the obtained 50% response value should also be reported if available.^28^

## Results

### Germination percentage, speed, and index

**Fig. 2:**
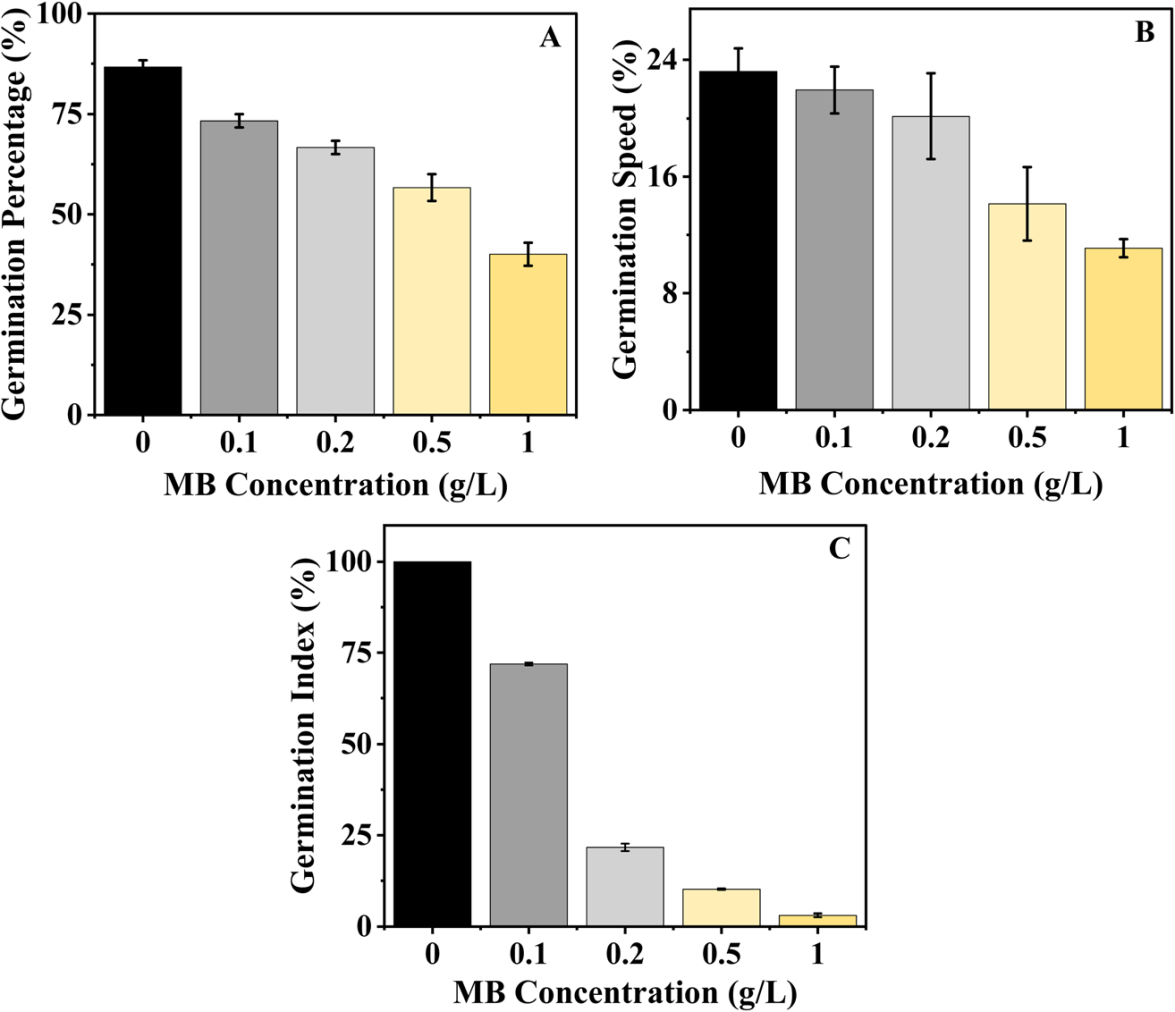
Effect of the concentration of methylene blue (MB) on germination parameters. (A) Germination percentage, (B) germination speed, and (C) germination index of *Brassica juncea* L. showed a decrease with an increase in the concentration of MB (from 0 to 1.0 g L^−1^).

Seed germination and seedling growth are two of the most important phases of the plant growth cycle and are highly sensitive to environmental stress. Seed germination is the primary physiological response of the seed to its environment. Germination percentage and seedling growth parameters are reliable tools for assessing environmental toxicity. In this study, the highest germination percentage for mustard was observed in the control group (86.66%). However, the germination percentage decreased with increasing MB concentration, reaching 73.34%, 66.66%, 56.67%, and 40% at 0.1, 0.2, 0.5, and 1.0 g L^−1^, respectively (Fig. 2). There was a decrease in germination rate with increasing concentration. The maximum rate of germination speed was recorded in the control group at 23.19 and gradually decreased to 21.93, 20.14, 14.13, and 11.08 at 0.1, 0.2, 0.5, and 1.0 g L^−1^, respectively (Fig. 2). The experiment shows that MB has an inhibitory effect on the rate of germination of the mustard plant, and the initial stages of germination of the plant are highly sensitive to the effects of dye stress. In the Germination Index (GI), which combines both the percentage of germination of seeds and their elongation, the use of MB decreased the GI value in a significantly defined way as the concentration of MB was increased. The highest GI value found was in the control treatment, meaning that under optimal conditions with no stress, a seedling would have better established itself. As seedlings were exposed to the MB, there was a continuous reduction in the GI value. The highest reductions of GI values were observed when the concentration of MB was at 0.2 g L^−1^ or greater, with the lowest GI value recorded when exposing seedlings to 1 g L^−1^. This is indicative of a complete suppression of both germination and the elongation of radicle growth (Fig. 2).

The decline in GI indicates that MB is affecting not just the number of seeds germinating, but also the overall development of the root early on, and therefore, compromising the overall vigour of the seedling.

### Seedling vigour index

**Fig. 3:**
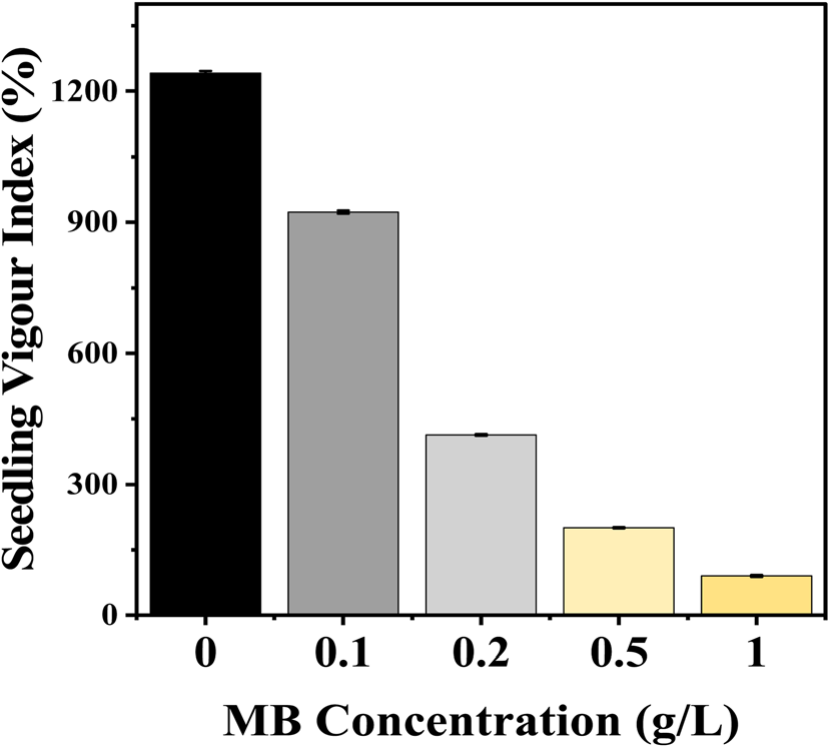
Effect of methylene blue (MB) concentration on seedling vigour index (SVI). The figure depicts an increase in the concentration of methylene blue (ranging from 0 to 1.0 g L^−1^), which led to a significant decrease in seedling vigour index.

Seedling growth measurements showed that the seedling vigour index (SVI) decreased significantly in mustard plants with increasing levels of MB concentration. Control (non-stressed) conditions resulted in maximum vigour index values (1241.33) compared to significantly lower vigour indexes after exposure to MB at 0.1 g L^−1^ (923.167), as well as at 0.2, 0.5, and 1.0 g L^−1^ (412.833, 200.667, and 90, respectively). The extent of vigour index reduction relative to increasing MB concentration shows that high levels of MB have a devastating effect on seedling growth and biomass accumulation (Fig. 3).

### Relative toxicity of seed germination

**Fig. 4:**
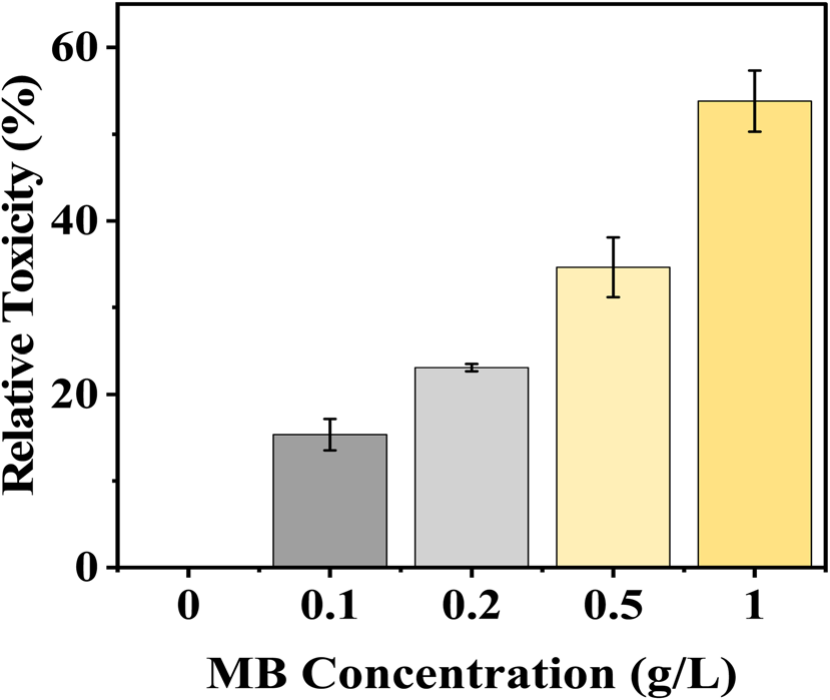
Effect of varying concentrations of methylene blue (MB) on relative toxicity (%) of *Brassica juncea* L. seedlings. The relative toxicity increased gradually with increasing concentration of MB (0–1.0 g L^−1^), showing a dose-dependent response. The higher concentration of MB showed greater toxicity, which means greater inhibition of seedling growth under methylene blue stress.

MB demonstrates a strong dose-dependent relative toxicity to mustard seed germination, with relative toxicity percentages increasing with increased dye concentration. A relative toxicity of 15.35% to mustard seeds occurred at a 0.1 g L^−1^ MB dye concentration, while a relative toxicity of 58.82% was noted at the highest concentration (1.0 g L^−1^). The percentage of toxicity increased gradually between 0.2 g L^−1^ and 0.5 g L^−1^ from 23.09% to 34.64%, respectively, showing that seed germination of mustard seeds was inhibited in a concentration-dependent manner (Fig. 4).

MB at a higher concentration will have greater toxicity relative to other substances and will have an adverse effect on the germination of mustard seeds. Inhibition of enzyme activation, alterations in membrane permeability, and lack of adequate water uptake to allow the embryo to grow or the radicle to protrude may be caused by MB due to the increased amount of toxicity present.

### Phytotoxicity

**Fig. 5:**
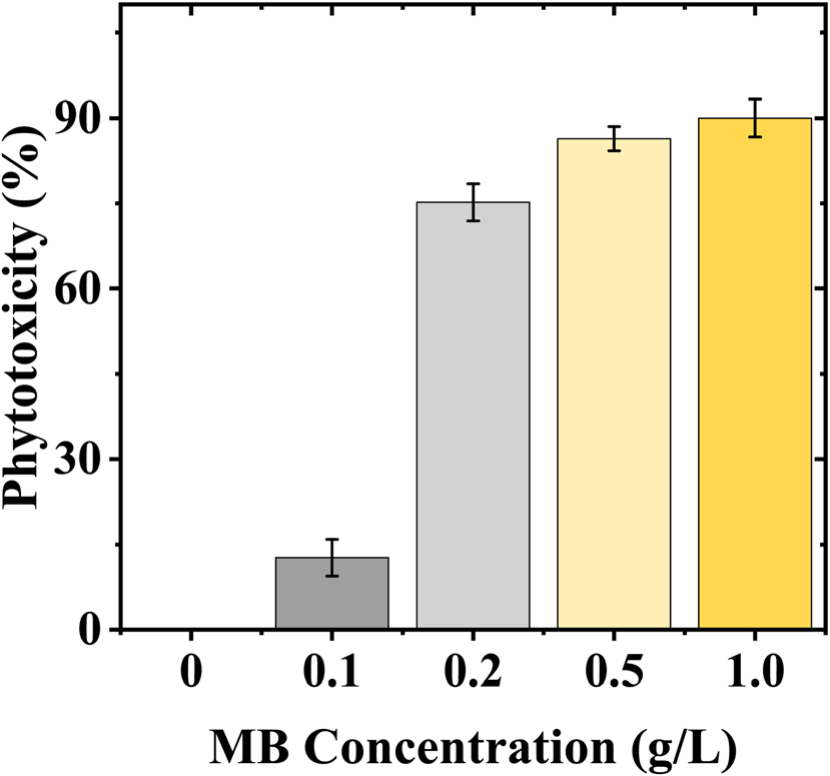
Effect of concentration of methylene blue (MB) on phytotoxicity (%) for *Brassica juncea* L. seedlings. Phytotoxicity increased with progressive increases in concentration (0 to 1.0 g L^−1^) of MB. Higher concentrations of MB exhibited a high degree of inhibition on mustard seedlings, thereby confirming the increased sensitivity of mustard seedlings to methylene blue exposure.

Phytotoxicity significantly increased with MB concentration. Control treatment had the lowest phytotoxicity (0%), while there was high inhibition of radicle elongation for mustard even at the lowest concentration (13.25% at 0.1 g L^−1^). There was a dramatic increase in phytotoxicity at 0.2 g L^−1^ (71.77%), and it remained at high levels (81.92%) at 0.5 g L^−1^ and increased to a maximum (93.34%) at 1.0 g L^−1^ for mustard. The high level of phytotoxicity indicates that the radicle elongation of mustard seeds is very sensitive to MB (Fig. 5).

### Shoot and root lengths

**Fig. 6:**
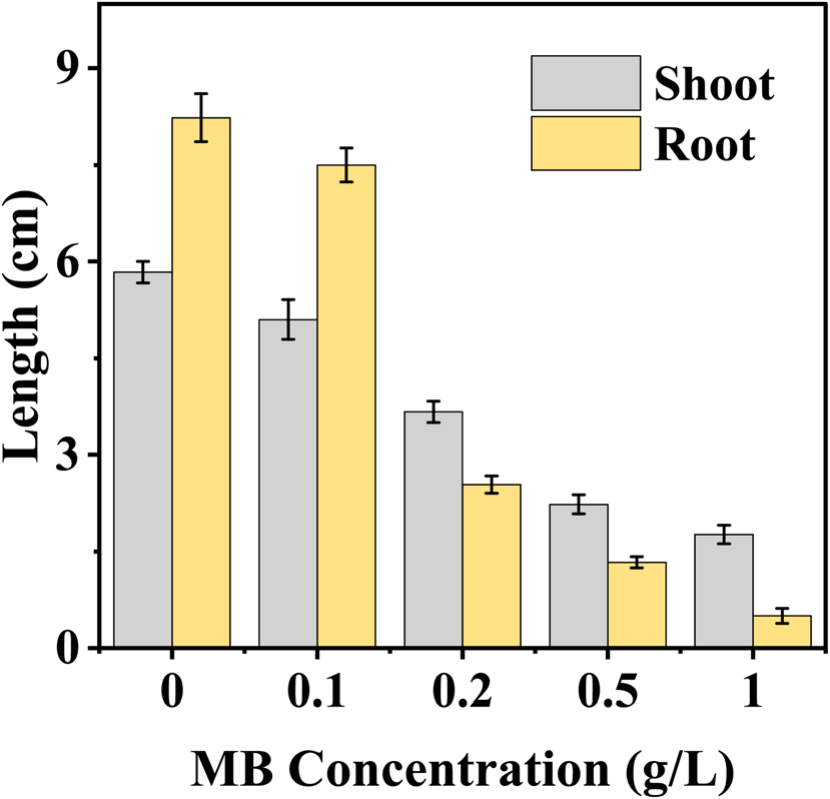
Effect of methylene blue (MB) concentration on the length of shoots and roots of *Brassica juncea* L. seedlings. The figure depicts that an increase in the concentration of methylene blue (0–1.0 g L^−1^) led to a decrease in the length of shoots and roots of the seedlings, with greater phytotoxicity in root growth.

Shoot and root length progressions were significantly impacted by MB concentrations on mustard seedling growth. The increase in MB concentrations resulted in a clear dose-dependent reduction in seedling parameters. Shoot length of mustard was significantly higher in control (5.8 cm) and followed by different MB concentrations 0.1, 0.2, 0.5, and 0.1 g L^−1^, the shoot lengths were decreased (5.1, 3.6, 2.2, and 1.8 cm), respectively. The root of control has the maximum radicle 8.2 cm; however, as the concentration of MB increased, progressive and linear reductions in radicle lengths were documented. At a concentration of 0.1 g L^−1^, the radicle was 7.5 cm; at 0.2 g L^−1^, it sharply decreased to 2.5 cm and continued sharply downward to 1.3 cm at 0.5 g L^−1^. At a 1.0 g L^−1^ concentration, radicle elongation was severely inhibited; the radicle measured 0.5 cm (Fig. 6).

### Fresh weight and dry weight

**Fig. 7:**
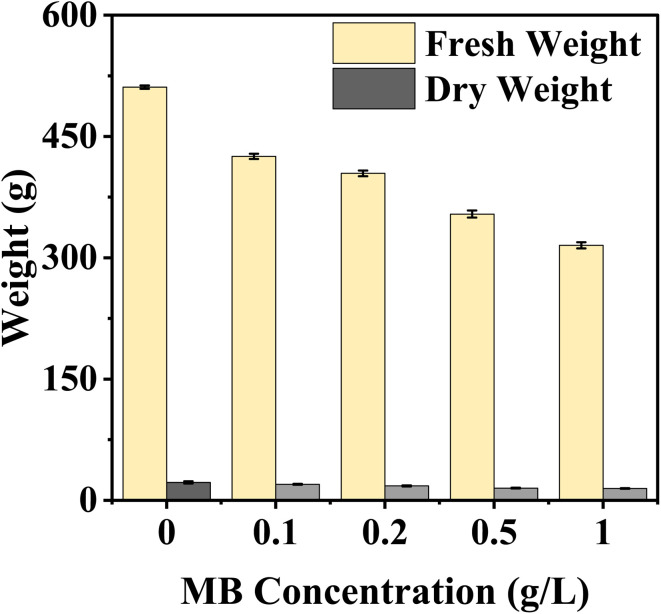
Effect of varying concentrations of methylene blue (MB) on fresh and dry weights of *Brassica juncea* L. seedlings. The figure shows that an increase in the concentration of methylene blue (0–1.0 g L^−1^) decreases the fresh and dry weights of the seedlings, which is an example of concentration-dependent phytotoxicity.

MB concentration negatively affected mustard seedlings' fresh and dry weight, with both values decreasing as exposure to the dye increased. The largest observed fresh and dry weights were measured from control seedlings, indicating that maximum biomass accumulation occurred under non-stressed conditions. However, fresh and dry weights continually decreased as seedlings were subjected to MB; therefore, when seedlings experienced a minimum dye concentration of 0.2 g L^−1^, fresh and dry weights decreased dramatically, and the lowest recorded values were found at a dye concentration of 1 g L^−1^(Fig. 7).

### Principal component analysis (PCA)

**Fig. 8:**
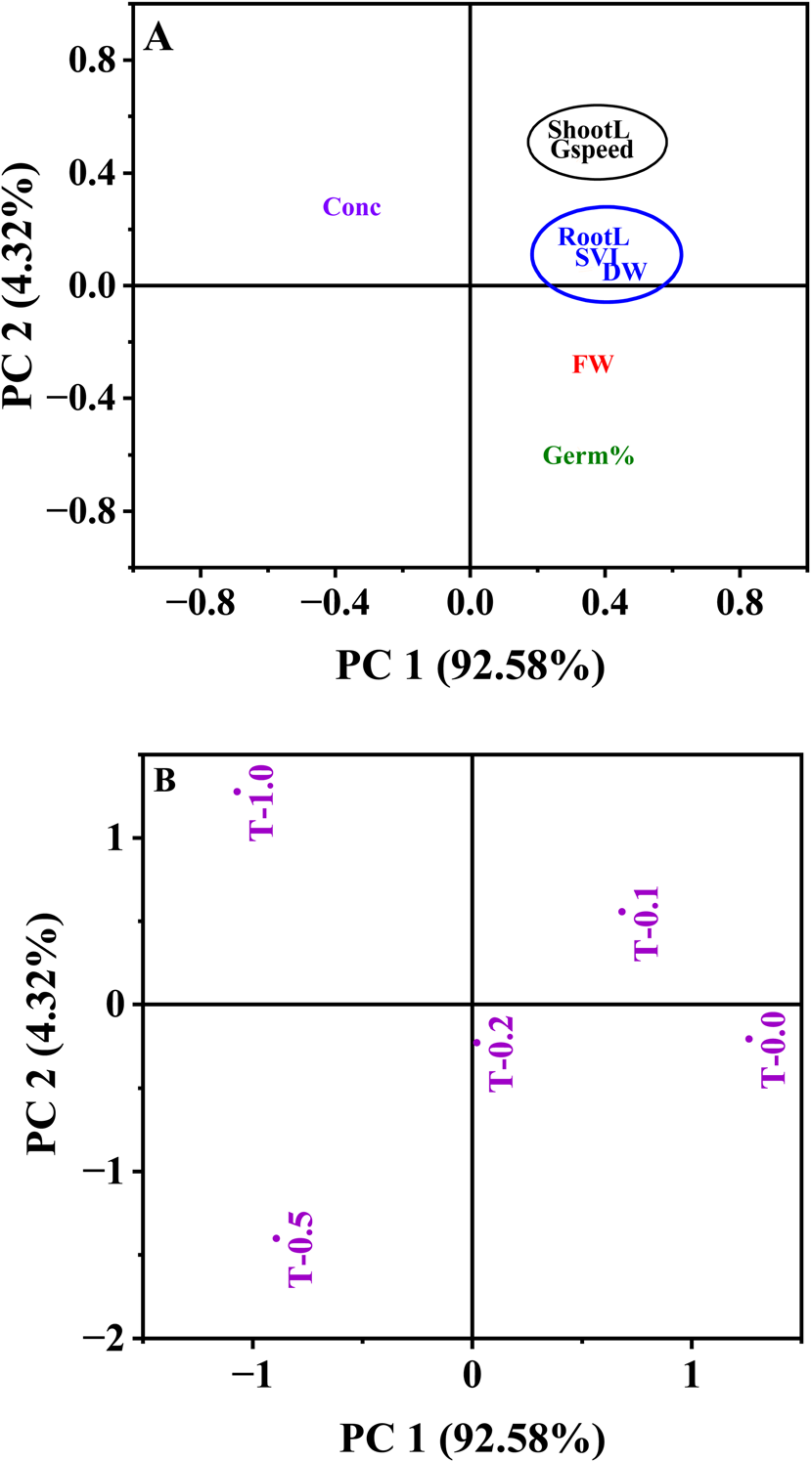
Principal component analysis (PCA) for understanding the relationship among germination and growth characteristics of *Brassica juncea* L. upon methylene blue treatment. (A) PCA loading plot showing the contribution of different variables, *i.e.*, germination percentage (Germ%), germination speed (Gspeed), root length (RootL), shoot length (ShootL), seedling vigour index (SVI), fresh weight (FW), dry weight (DW), and concentration (Conc) of methylene blue. (B) PCA score plot showing the clustering of different treatments (T-0.0, T-0.2, T-0.4, T-0.6, T-1.0 g L^−1^) of methylene blue. The variance explained by PC1 and PC2 is 92.58% and 4.32%.

We used PCA to assess how different levels of MB affect the development of mustard seedlings, to assess the multivariate responses of mustard seedlings to increasing levels of MB stress, and to identify which variables were mostly responsible for causing phytotoxicity. PCA separated the treatments by concentration of MB, with control seedlings and seedlings receiving low levels of MB on one end of the toxicity gradient (PC1, 92.58% total variance) and seedlings exposed to higher levels of MB on the other end of the gradient (PC1, opposite ends of the gradient). The loading plot suggested that MB concentration had a negative association with PC1, while root length, shoot length, SVI, and both fresh and dry weights of the seedlings were positively associated with PC1. PCA further indicates that increasing levels of MB correlate with decreased germination and seedling growth characteristics. In total, PC2 accounted for only 4.32% of the variation by contributing only a small amount to the overall variance. In total, the results of this PCA suggest that an elevated level of MB is likely to adversely affect mustard seedling development and that germination and growth-related traits are the main indicators of phytotoxicity (Fig. 8).

### Analysis of MB toxicity

**Fig. 9:**
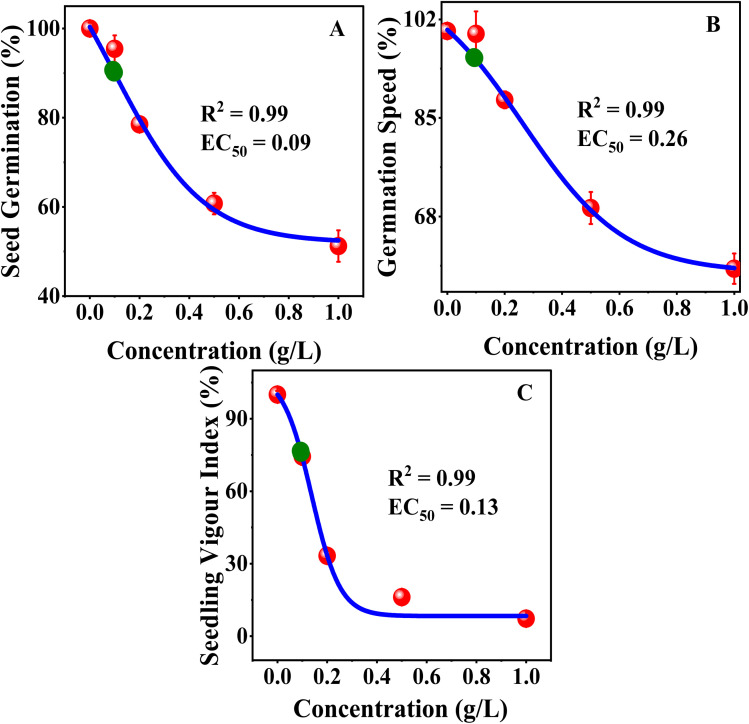
Dose–response curves for *Brassica juncea* L. Effects of methylene blue (MB) on (A) seed germination, (B) germination rate, and (C) seedling vigour index. Nonlinear Boltzmann fitting was used to determine EC_50_. Increasing MB concentration caused a corresponding decline across all three parameters, indicating a phytotoxic effect.

**Fig. 10:**
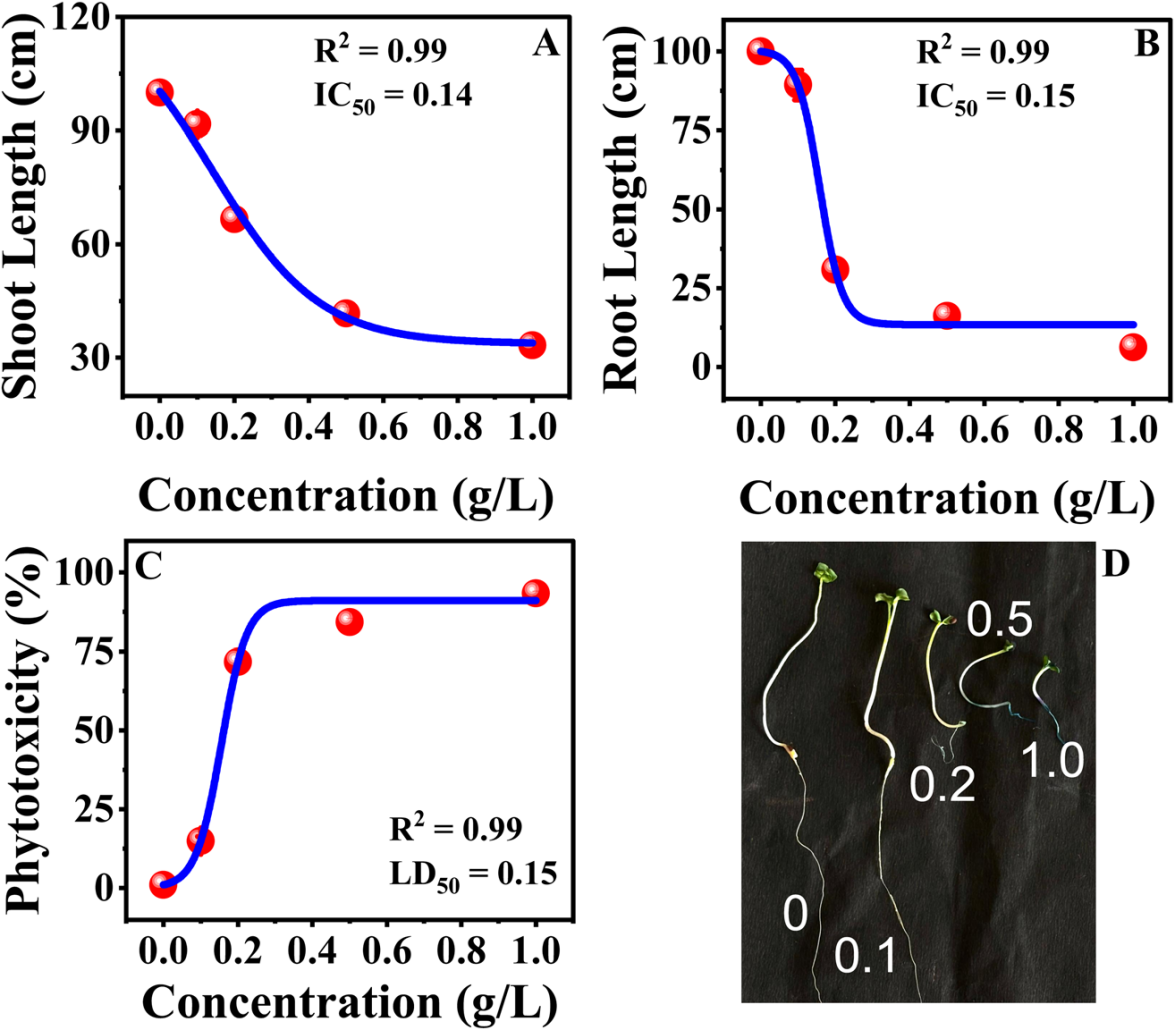
Toxicity evaluations of methylene blue on *Brassica juncea* L. This figure represents the IC_50_ and LD_50_ value analysis for (A) shoot length and (B) root length. It also details the LD_50_ value analysis for (C) phytotoxicity and (D) growth of seventh-day plants across different MB concentrations.

The dose–response analysis was conducted using a Boltzmann sigmoidal nonlinear fit, which indicated that the sensitivity of mustard seedlings to MB was different for physiological traits. The value of EC_50_ was employed for germination-related traits because these parameters measure the effective reduction in seed performance as the concentration of dye increases (Fig. 9). The value of IC_50_ was employed for root growth and shoot growth because these parameters measure the inhibition of growth. The value of LD_50_ was employed for phytotoxicity because this parameter measures a 50% toxic response (Fig. 10). The sigmoidal curves obtained from the nonlinear fit indicated that germination was more tolerant to MB, as a higher concentration was necessary to achieve a 50% response, whereas seedling vigour index and root growth traits showed a response to a lower concentration, making these more sensitive. The sharper decline in the root growth and phytotoxicity curves indicates a more rapid inhibition response over a relatively narrower concentration range. The nonlinear dose–response analysis confirms that root growth and seedling vigour index are more sensitive to MB, whereas germination is more tolerant. These findings support the conclusion that MB primarily inhibits seedling establishment through a potent inhibition of root growth, leading to a loss in seedling performance.

### Hierarchical cluster analysis (HCA)

**Fig. 11:**
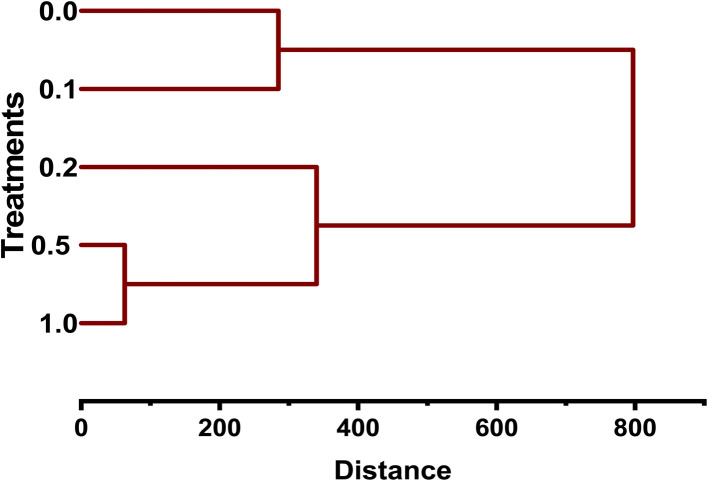
Hierarchical cluster dendrogram for the grouping of *Brassica juncea* L. treatments with methylene blue. The lower concentrations (0.0 and 0.1 g L^−1^) were grouped together, whereas the higher concentrations (0.5 and 1.0 g L^−1^) formed another group. The treatment with 0.2 g L^−1^ was found to be intermediate in similarity.

Cluster analysis (CA) was conducted to investigate the similarity amongst treatments of MB regarding how they affect seedling growth and germination of mustard. The dendrogram showed distinct patterns among groups, with treatments affecting growth responses (GER, RL, SL, and SVI) in a concentration-dependent manner. The treatments for control and 0.1 g L^−1^ showed close clustering, indicating that the means of GER, RL, SL, and SVI of the seedlings did not vary significantly from each other in terms of low-level conditions. This suggests that lower MB concentrations would result in minimal physiological disturbance. The treatments at concentrations of 0.5 and 1.0 g L^−1^ formed a separate cluster, showing considerable similarity in their effects on seedling growth inhibition. There was a substantial decrease in all growth parameters, with greater phytotoxicity at higher MB concentrations; this finding aligns with dose-dependent responses in seedlings. The treatment at a concentration of 0.2 g L^−1^ occupied an intermediate position between the two previously described clusters, which were distinct in similarity (Fig. 11).

### Heatmap analysis of multivariate relationships

**Fig. 12:**
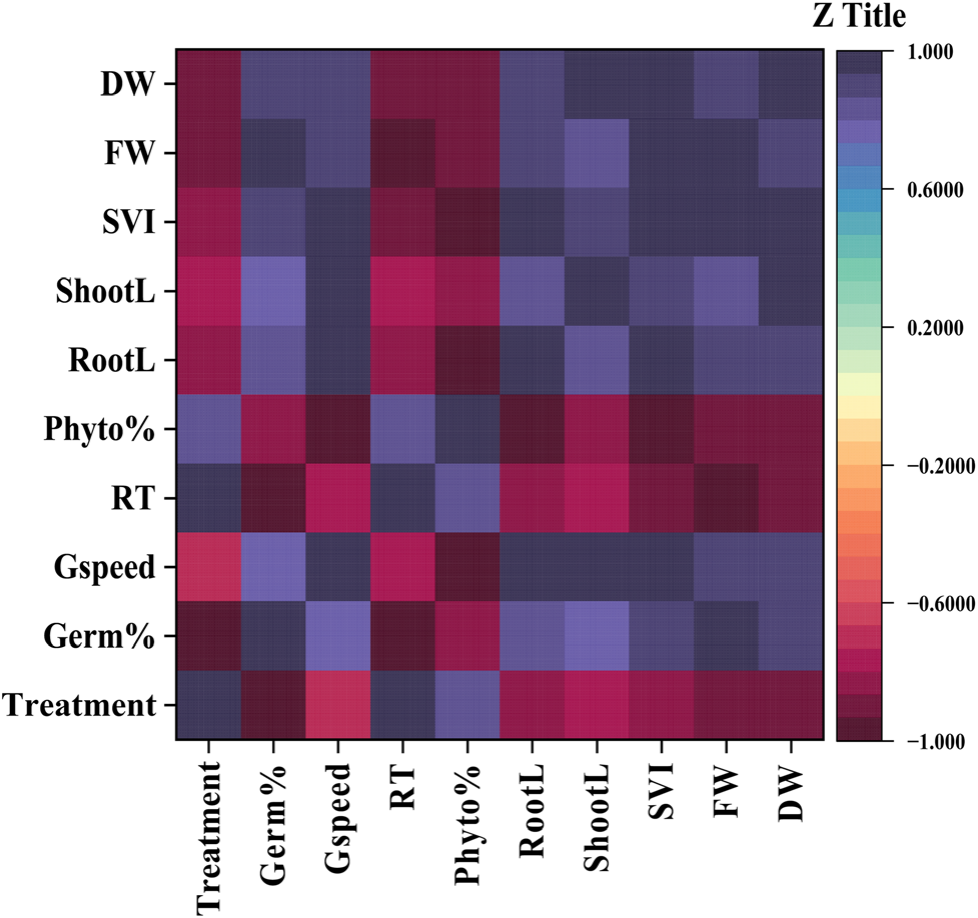
Heatmap correlation matrix showing the correlation between germination and seedling growth parameters of *Brassica juncea* L. upon treatment with methylene blue (MB). Variables included treatment concentration, germination percentage (Germ%), germination speed (Gspeed), relative toxicity (RT), phytotoxicity (Phyto%), root length (RootL), shoot length (ShootL), seedling vigour index (SVI), fresh weight (FW), and dry weight (DW). Pearson correlation coefficient values ranging from −1 (red, negative correlation) to +1 (blue, positive correlation) are depicted in different colors. Growth parameters exhibited strong positive correlations among themselves and negative correlations with treatment concentration, relative toxicity, and phytotoxicity, suggesting that MB inhibited mustard seedling growth in a dose-dependent manner.

This heatmap shows the Pearson correlation matrix, examining the relationship between MB concentration and various measured germination and growth parameters in mustard plants. The gradient of colors in the heatmap shows the strength of each association, from strongly negative (−1) to strongly positive (+1). The analysis has revealed 2 distinct clusters of variables based upon their correlations to either toxicity or growth. For example, the concentration of MB shows a strong positive correlation with the relative toxicity and phytotoxicity percentages. It has been shown that as the dye concentration increases, so do the toxic effects of the dye. Conversely, the treatment concentration shows a strong negative correlation with germination percentage, germination speed, root length, shoot length, seedling vigour index (SVI), fresh weight, and dry weight. This shows that the development of the early seedling has been suppressed in a dose-dependent manner (Fig. 12).

## Discussion

To comprehensively evaluate the phytotoxicity of methylene blue (MB) on *Brassica juncea* L., this study employed a concentration ranging up to 1.0 g L^−1^. While typical environmental concentrations of synthetic dyes in treated aquatic systems are generally lower, ranging from 10 to 250 mg L^−1^ (0.01 to 0.25 g L^−1^), there have been reports where the dye concentration in the watercourses can reach 1.0 g L^−1^ or even more.^29^ Nonetheless, the higher concentrations employed here were specifically selected to model extreme ecological scenarios and establish a complete dose–response curve.^6^ Such elevated concentrations are highly relevant in the context of unregulated, small-scale textile industries in developing nations, where untreated effluents are frequently discharged directly into local water bodies or utilized for crop irrigation. Furthermore, because synthetic dyes like MB are environmentally persistent, repeated irrigation with even low-concentration effluents can lead to the localized accumulation of these compounds within the agricultural soil matrix. This continuous deposition exposes crop root systems to much higher effective concentrations than what is measured in the water alone.^30^

Under these simulated exposure scenarios, our results demonstrate a severely enhanced phytotoxic response, with increasing concentrations of MB systematically inhibiting the seed germination and early seedling development of mustard. As MB concentration increased, all the parameters studied, including % germination, germination speed, and seedling vigour index, were negatively affected and decreased as MB concentration increased, whereas relative toxicity (RT) and phytotoxicity increased; it is obvious that MB has inhibited germination and seedling growth of mustard, thereby confirming that seed germination is highly sensitive to the stress of synthetic dye. Synthetic dyes such as MB are known to interfere with cellular metabolism by generating oxidative stress and altering membrane permeability, thereby affecting early physiological events during seed germination.^7^ Synthetic dyes like MB have been known to induce ROS generation, resulting in oxidative damage to cellular membrane structures, proteins, and nucleic acids, affecting metabolic processes related to germination.^7,31^ This is because the germination index combines both the germination process and root elongation. Therefore, it is a more holistic measure of seedling performance under stress conditions.^32,33^

The reduction in germination may be associated with interference in metabolic and physiological processes involved in seed germination. Inhibition of seed germination has also been reported under salt, heavy metal, and industrial effluent stress in various plant species.^34,35^ Higher concentrations of MB can inhibit seed germination by affecting hormonal balance, including auxins, which handle seed germination.^36^ The reduction in germination speed may be due to delayed metabolic activation, possibly associated with a reduced permeability of seed membranes, which are responsible for enzymatic activities and the mobilization of stored food reserves for seed germination.^34,35,37^ Delayed germination has also been reported under different types of stress in crop plants as well as allelopathic interactions.^38,39^ Nevertheless, as opposed to some studies on industrial effluent where low concentrations stimulate growth due to nutrient enrichment, MB has shown a strictly inhibitory effect at all concentrations, suggesting a direct toxic mechanism rather than nutrient enrichment.^6^

This marked reduction in GI values with increasing MB concentration is of particular interest, since GI accounts not only for the percentage of successful germination but also for radical growth. It is a more sensitive indicator of phytotoxicity than germination percentage. The efficacy of GI as an indicator of stress response in seeds is now widely accepted.^33^ From the results of the present study, the sharp decline in GI values at and above 0.2 g L^−1^, Fig. 1, suggests not just the inhibition of seed germination but also the impairment of root growth. This impairment of root growth is likely because of damage to the cell membrane, reduction of water uptake, and oxidative stress, which impair embryo expansion.^31,40^ Similar phytotoxic effects of effluents containing dyes have been reported for red amaranth, rice, and lady's finger.^10,24^ The growth of both the radicle and the plumule was inhibited. Radicle growth, however, was more sensitive than plumule growth. This is in line with the general observation that the roots are the first to be affected by dissolved toxins in the soil water. The roots often show a stronger response than the shoots.^41^ Root tissue is particularly susceptible to the effects of contaminants because it is the first tissue to come into contact with the contaminants. This disrupts membrane integrity, nutrient uptake, and cell elongation processes.^41^ Such inhibition is often associated with mitochondrial dysfunction and reduced ATP production, which limits energy availability for rapid cell expansion in growing root tissues.^31^ The inhibition of root growth is likely due to reduced respiration, decreased ATP production, changes in nutrient uptake, damage to the cell membrane, and effects on cell division and elongation.^41,42^ The absence of any stimulation of growth, even at the lowest concentration of the effluent, is of particular interest. This contrasts with the effects of some industrial effluents, where low concentrations of the effluent stimulated seedling growth through nutrient enrichment.^43^ The absence of any nutrient effect of the effluent in the present study is likely due to the effluent being a cationic dye.

The continuous increase in relative toxicity and phytotoxicity with the increase in concentration of MB also points to the existence of a dose–response relationship. Concentrations above 0.2 g L^−1^ induced major inhibition, and those above 0.5 g L^−1^ induced severe toxicity. These trends have been observed for crop seeds exposed to industrial wastewater and synthetic dye mixtures.^6,44,45^ The high levels of phytotoxicity observed in the present study suggest that MB has significant effects on radicle elongation, which could involve the induction of oxidative stress, alterations in water relations, and the inhibition of enzyme activities.^31,41^ The reduction in fresh and dry biomass is also in line with the above-mentioned mechanism. The roots remained in direct contact with the dye solution throughout the experiment, and their growth inhibition would limit nutrient and water uptake, thereby limiting total biomass accumulation. Dose–response analysis has shown that MB has a stage- and organ-specific effect on mustard seedlings. Higher EC_50_ values in germination imply a degree of tolerance in seed activation, but lower IC_50_ values in germination rate and SVI imply early effects on seedling metabolism. The high sensitivity of SVI further supports its use as a reliable biomarker, as it reflects both germination efficiency and seedling growth performance under stress conditions.^33^ LD_50_ analysis has shown that, again, root length and phytotoxicity are the most sensitive, emphasizing the importance of the roots in the seedling. Direct interaction with the roots is likely to interfere with membrane structure, water uptake, and cell division. Secondary effects on stem growth suggest lesser importance, again emphasizing the primary effect of MB on seedling establishment *via* the roots. This is consistent with earlier studies that have shown that synthetic dyes have a substantial impact on early plant development by causing oxidative stress, thereby interfering with physiological processes.^7,46^ It can be concluded that MB exerts significant toxicity during the initial development of seedlings and poses a major threat to crop yield if introduced into the system through the medium of wastewater containing the dye.^33^

While the main data from this work is a clear quantification of the severe phenotypic and developmental inhibition induced by MB (*i.e.* reduced radicle elongation, biomass decline, and suppressed germination), to understand the root cause of this toxicity, it is necessary to combine our empirical observations with well-established molecular mechanisms from the literature. The drastic reduction of seed germination and seedling vigour in our bioassays is not a mere physical blocking effect but a serious disruption of crucial early physiological cascades, based on recent ecotoxicological studies, which are outlined subsequently. For instance, literature indicates that in response to dye exposure, MB-induced oxidative stress causes an overproduction of ROS that attacks the seed and root cell ‘membranes’ lipid bilayers. Lipid peroxidation damages membrane integrity, causing electrolyte leakage and the inability of seeds to maintain the osmotic potential required for adequate water imbibition, which is a prerequisite for the initiation of germination.^41,42^ Moreover, the build-up of toxic dye molecules in the seed tissues strongly suppresses the activity of important hydrolytic enzymes such as alpha-amylase, which are responsible for breaking down stored starch into the soluble sugars needed to power the developing embryo. Thus, the observed inhibition of radicle and plumule elongation is a physiological result of both nutrient starvation due to inhibition of metabolic enzymes and cellular damage due to ROS-mediated oxidative bursts.^30,40^

In order to gain insight into the mechanism of the biological effects observed above, it is critical to examine the chemical structure and characteristics of methylene blue. As a synthetic cationic thiazine dye (C_16_H_18_ClN_3_S), MB displays a highly localized positive charge across its heterocyclic nitrogen–sulfur aromatic rings. The positive charge associated with MB leads to highly electrostatic interactions with the negatively charged groups found in plant cells, such as the carboxylates of pectins and the hydroxyls of cellulose that make up the cell wall and plasma membrane.^47^ Electrostatic interactions between the cationic dye and components of the cell walls interfere not only with the cellular intake of nutrients but also lead to permanent changes in membrane permeability. In addition, the planar and aromatic molecular structure of MB allows the compound to act as an effective redox mediator within the cellular environment. In particular, MB molecules can act as a non-catalytic mediator of ROS production by acting as an electron acceptor.^31^ As an electron acceptor, MB rapidly transfers electrons to oxygen, thus promoting local overproduction of ROS, including superoxide and singlet oxygen radicals.^31^ Consequently, the resulting chemically induced oxidative stress results in the oxidation of lipids, inactivation of enzymes responsible for plant metabolism, inhibition of mitochondrial activity, and loss of ATP production.^48,49^ As documented in established literature, these cellular events ultimately arrest plant growth, perfectly aligning with the rapid, dose-dependent collapse of radicle elongation and seedling vigour quantified in our IC_50_ and LD_50_ models.

Multivariate approaches such as PCA are particularly useful in phytotoxicity studies because they allow simultaneous evaluation of multiple physiological parameters and help identify the most sensitive indicators of stress.^28^ This approach used in this study provides useful mechanistic insight beyond dose–response descriptions. Principal Component Analysis (PCA) clearly separated control/low dose treatments from higher MB concentrations, with strong negative values for germination and growth parameters, and strong positive values for phytotoxicity and relative toxicity. The high loading and strong collinearity of radicle length, Seedling Vigour Index (SVI), and biomass along the first axis (PC1) in the Principal Component Analysis (PCA) indicated that these parameters are the main drivers of the physiological variance observed. This statistical grouping suggests that the primary physiological bottleneck under MB stress is radicle elongation, making radicle length and SVI, in effect, the most sensitive, early-warning biomarkers of cationic dye phytotoxicity.^30^ Moreover, the HCA proves to have added value in that it classifies the gradient of MB exposure into different physiological regimes, instead of a linear decrease. The clustering analysis of various MB treatments indicates a major physiological tipping (*e.g.*, the switch from the 0.2 g L^−1^ to the 0.5 g L^−1^ concentrations). Below this threshold, basal plant defense mechanisms such as localized antioxidant responses may, to some extent, counteract dye-induced oxidative damage. However, the separate clustering of higher concentration treatments shows a systemic collapse of these defenses, leading to severe and irreversible toxicity and arrested cell division.^40,41^ Complementing the PCA and HCA, the heatmap analysis provides a high-resolution visualization of this dose-dependent physiological collapse. The intensity matrix explicitly illustrates the downregulation of vital growth parameters, coinciding with the sharp upregulation of phytotoxicity indices at higher concentrations. Strong positive intercorrelations among growth parameters were evident in the heatmap, alongside strong negative correlations between these parameters and toxicity. In summary, while the univariate dose–response models do well to confirm the overall concentration-dependent toxicity of MB, the integrated multivariate framework adds important additional insight by moving the analysis from descriptive to predictive. This approach mathematically separates radicle elongation and SVI as the basic causative factors of variance (*via* PCA) and pinpoints the precise point at which basal plant defenses systematically break down (*via* HCA and heatmap), rather than considering all physiological decreases equal. Thus, the main added value of the multivariate approach is to retrieve a sensitivity hierarchy from classical bioassay data, which turns basic physiological measurements into specific early-warning biomarkers and non-linear ecological tipping points for robust agricultural risk assessment.

## Conclusion

In this study, we have demonstrated that MB causes significant phytotoxic effects on the germination and seedling growth of mustard plants (*Brassica juncea* L.). With an increase in dye concentration, the percentage of germination, germination rate, germination index, root length, shoot length, seedling vigor index, and biomass were significantly reduced, whereas relative toxicity and phytotoxicity were substantially increased. Among the growth parameters, root length and seedling vigor index were identified as the most responsive traits to MB toxicity, as confirmed by multivariate statistical analyses. In this study, principal component analysis, hierarchical cluster analysis, and heatmap correlation results showed a strong negative correlation between MB concentration and growth-related traits, indicating that root growth is a major factor in overall seedling phytotoxicity. It is evident that mustard plants in the early growth stages are highly susceptible to MB toxicity. Therefore, the discharge of dye-containing industrial wastewater into agricultural environments must be well-regulated to avoid ecological hazards.

## Author contributions

Lopamudra Subudhi: formal analysis, investigation, data curation, writing – original draft, writing – review & editing, visualization. Alok Kumar Panda: conceptualization, methodology, software, validation, formal analysis, investigation, resources, data curation, writing – original draft, writing – review & editing, visualization, supervision, project administration and Shibani Mohapatra: conceptualization, methodology, formal analysis, investigation, resources, data curation, writing – original draft, writing – review & editing, visualization, supervision, project administration, funding acquisition.

## Conflicts of interest

The authors declare that there are no conflicts of interest regarding the publication of this manuscript.

## Data Availability

The data supporting the findings of this study are available within the article. Additional data related to this study are available from the corresponding author upon reasonable request.
